# Factors Associated With Opioid Overdose After an Initial Opioid Prescription

**DOI:** 10.1001/jamanetworkopen.2021.45691

**Published:** 2022-01-28

**Authors:** Scott G. Weiner, Sanae El Ibrahimi, Michelle A. Hendricks, Sara E. Hallvik, Christi Hildebran, Michael A. Fischer, Roger D. Weiss, Edward W. Boyer, Peter W. Kreiner, Dagan A. Wright, Diana P. Flores, Grant A. Ritter

**Affiliations:** 1 Department of Emergency Medicine, Brigham and Women’s Hospital, Boston, Massachusetts; 2 Division of Research and Evaluation, Comagine Health, Portland, Oregon; 3 School of Public Health, Department of Epidemiology and Biostatistics, University of Nevada, Las Vegas; 4 Section of General Internal Medicine, Boston Medical Center, Boston University School of Medicine, Boston, Massachusetts; 5 Harvard Medical School, Boston, Massachusetts; 6 McLean Hospital, Belmont, Massachusetts; 7 Schneider Institutes for Health Policy, Heller School for Social Policy and Management, Brandeis University, Waltham, Massachusetts; 8 OCHIN Inc, Portland, Oregon

## Abstract

**Importance:**

The opioid epidemic continues to be a public health crisis in the US.

**Objective:**

To assess the patient factors and early time-varying prescription-related factors associated with opioid-related fatal or nonfatal overdose.

**Design, Setting, and Participants:**

This cohort study evaluated opioid-naive adult patients in Oregon using data from the Oregon Comprehensive Opioid Risk Registry, which links all payer claims data to other health data sets in the state of Oregon. The observational, population-based sample filled a first (index) opioid prescription in 2015 and was followed up until December 31, 2018. Data analyses were performed from March 1, 2020, to June 15, 2021.

**Exposures:**

Overdose after the index opioid prescription.

**Main Outcomes and Measures:**

The outcome was an overdose event. The sample was followed up to identify fatal or nonfatal opioid overdoses. Patient and prescription characteristics were identified. Prescription characteristics in the first 6 months after the index prescription were modeled as cumulative, time-dependent measures that were updated monthly through the sixth month of follow-up. A time-dependent Cox proportional hazards regression model was used to assess patient and prescription characteristics that were associated with an increased risk for overdose events.

**Results:**

The cohort comprised 236 921 patients (133 839 women [56.5%]), of whom 667 (0.3%) experienced opioid overdose. Risk of overdose was highest among individuals 75 years or older (adjusted hazard ratio [aHR], 3.22; 95% CI, 1.94-5.36) compared with those aged 35 to 44 years; men (aHR, 1.29; 95% CI, 1.10-1.51); those who were dually eligible for Medicaid and Medicare Advantage (aHR, 4.37; 95% CI, 3.09-6.18), had Medicaid (aHR, 3.77; 95% CI, 2.97-4.80), or had Medicare Advantage (aHR, 2.18; 95% CI, 1.44-3.31) compared with those with commercial insurance; those with comorbid substance use disorder (aHR, 2.74; 95% CI, 2.15-3.50), with depression (aHR, 1.26; 95% CI, 1.03-1.55), or with 1 to 2 comorbidities (aHR, 1.32; 95% CI, 1.08-1.62) or 3 or more comorbidities (aHR, 1.90; 95% CI, 1.42-2.53) compared with none. Patients were at an increased overdose risk if they filled oxycodone (aHR, 1.70; 95% CI, 1.04-2.77) or tramadol (aHR, 2.80; 95% CI, 1.34-5.84) compared with codeine; used benzodiazepines (aHR, 1.06; 95% CI, 1.01-1.11); used concurrent opioids and benzodiazepines (aHR, 2.11; 95% CI, 1.70-2.62); or filled opioids from 3 or more pharmacies over 6 months (aHR, 1.38; 95% CI, 1.09-1.75).

**Conclusions and Relevance:**

This cohort study used a comprehensive data set to identify patient and prescription-related risk factors that were associated with opioid overdose. These findings may guide opioid counseling and monitoring, the development of clinical decision-making tools, and opioid prevention and treatment resources for individuals who are at greatest risk for opioid overdose.

## Key Points

**Question:**

What factors are associated with an increased risk for opioid overdose after the initial opioid prescription to a previously opioid-naive individual?

**Findings:**

In this cohort study of 236 921 individuals who received a first opioid prescription, 667 experienced an incident opioid overdose. Patient risk factors included being aged 75 years or older, being male, receiving Medicaid or Medicare Advantage coverage, having a comorbid substance use disorder or depression, and having medical comorbidities. Prescription-related risk factors included an initial prescription of oxycodone or tramadol, concurrent use of benzodiazepines, and filling opioid prescriptions from 3 or more pharmacies.

**Meaning:**

Findings from this study suggest that several patient- and prescription-related risk factors are associated with opioid overdose; prescribers, researchers, policy makers, and insurers can apply this information to guide opioid counseling and monitoring, develop clinical decision-making tools, and provide additional opioid prevention and treatment resources to individuals who are at greatest risk for opioid overdose.

## Teaser

This cohort study investigates patient- and prescription-related factors associated with opioid-related fatal or nonfatal overdose among opioid-naive individuals receiving an initial opioid prescription.

## Introduction

Opioid medications remain a mainstay of treatment of severe pain. In the setting of the modern opioid overdose and death epidemic, use of such medications has decreased, but there were still 168.9 million opioid prescriptions in the US in 2018.^1^ Each prescription of an opioid to a previously opioid-naive patient creates the potential for the development of chronic opioid use and opioid use disorder.^2^ For this reason, multiple entities and states have produced opioid prescribing guidelines, such as the Centers for Disease Control and Prevention Guideline for Prescribing Opioids for Chronic Pain in 2016 and the Oregon Acute Opioid Prescribing Guidelines in 2018.^3,4^ The surgical literature has declared opioid dependence to be a never-event (along with use disorder and overdose)^5^ and as the most common surgical complication,^6^ affecting approximately 5% to 7% of patients who started a new episode of opioid use.^7^

An association exists between the characteristics of a patient’s first opioid prescription and long-term use. Shah et al^8^ discovered that the number of days’ supply of the initial prescription was directly associated with the development of long-term use. Deyo et al^9^ found that greater morphine milligram equivalents (MMEs) of the initial prescription were associated with increased likelihood of developing long-term use. Previous work with the Ohio prescription drug monitoring program (PDMP) database found that different prescriber specialties had different rates of long-term use by patients^10^ likely because the indications of opioid use and underlying patient factors create different risks for long-term use.

Although chronic opioid use is an undesirable outcome, the most substantial harms from a new episode of prescription opioid use are overdose and death. Unfortunately, the ability to link prescribing decisions to specific outcomes has been hampered by data source limitations. Prescription drug monitoring programs describe the prescription filling patterns of patients with long-term use but do not contain data about drug indications, comorbid conditions, the patient’s environment, or intervening outcomes, such as opioid use disorder or overdose. Administrative claim files are useful because they capture prescriptions that are covered by insurance but may be incomplete if the prescriptions are paid in cash or if patients change insurers. In addition, the most serious outcome of interest, opioid-related death, is often found not in PDMP or insurance data but in vital records.

To address data source limitations and provide a more comprehensive analysis of risk factors after the initiation of opioid therapy, we combined claims data with several public health data sets (including All Payer All Claims Data [APCD], vital records, PDMP, and hospital discharge data) to create the Oregon Comprehensive Opioid Risk Registry.^11^ Using the Comprehensive Opioid Risk Registry, we constructed a retrospective cohort of opioid-naive patients who received an initial opioid prescription to assess the patient factors and early time-varying prescription-related factors associated with opioid-related fatal or nonfatal overdose. We hypothesized that some patient factors (eg, insurance type and high disease burden) and prescription factors in the first 6 months after opioid initiation (eg, high doses, and opioid and benzodiazepine overlap) are associated with increased risk of opioid overdose.

## Methods

Activities for this cohort study were approved by the Mass General Brigham Human Research Committee, which waived the patient consent requirement because this study posed minimal risk to participants, and the research could not practicably be conducted without the waiver. We followed the Strengthening the Reporting of Observational Studies in Epidemiology (STROBE) reporting guideline.

### Data Sources and Preparation

In this retrospective cohort study, we used the 2013 to 2018 claims data from the voluntary APCD of Oregon, which is linked by patient identifier to several public health data sets, including the Oregon Vital Records death certificates, the Oregon PDMP, and the Oregon hospital discharge database. Most commercial and Medicare Advantage (which is managed by commercial insurance programs) plans in the state report into the APCD, covering approximately 80% of commercially insured individuals; data on all individuals with Medicaid coverage are also reported to the APCD. Using linkage and deduplication software (fastLink in R; R Foundation for Statistical Computing),^12^ we probabilistically linked each public health data set to the APCD by individual beneficiary’s first name, last name, and birthdate. The cohort was derived from approximately 3.6 million patients in the APCD with valid Oregon addresses. The linkage methods are described in detail elsewhere.^11^ Because of data use agreement restrictions, we excluded patients with Medicare fee-for-service from the linkage.

### Study Sample

The study sample included opioid-naive adults aged 18 to 100 years from the APCD enrollment file who had 1 or more opioid prescriptions filled in 2015 based on the PDMP. We selected the first prescription in 2015 as the index prescription and considered the following 6 months to be the index period. To be considered opioid naive, individuals must not have had more than 1 index prescription; an opioid use disorder–related buprenorphine formulation as the index prescription or in the index period; or any opioid prescriptions, opioid-related hospitalizations (eTable 1 in the Supplement), or emergency department visits in the 12 months before the index prescription dispense date. Patients with missing sex information were also excluded. Insurance coverage was captured on an annual calendar basis, and continuous enrollment was required in 2015 with an allowed 90 days’ gap (Figure). Patients were censored if they had an opioid overdose event, died of other causes, lost insurance coverage, or were event-free at the end of follow-up (December 31, 2018), whichever occurred first.

**Figure.**
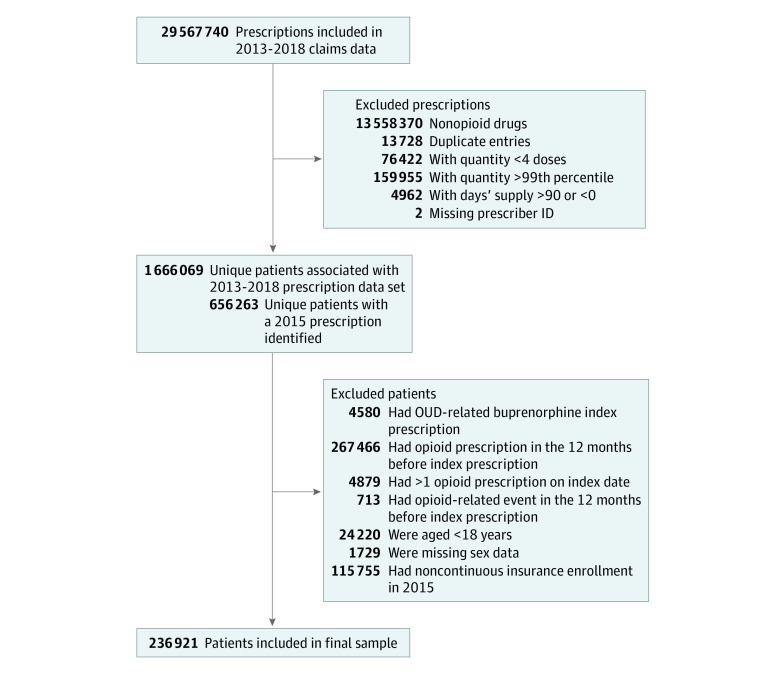
Prescription- and Patient-Level Exclusions OUD indicates opioid use disorder.

### Risk Factors

#### Patient Demographics

We categorized age into age bands (18-24, 25-34, 35-44, 45-54, 55-64, 65-74, or ≥75 years). Race and ethnicity were obtained from the APCD enrollment files and had the following categories: Hispanic, non-Hispanic Asian or Pacific Islander, non-Hispanic Black (hereafter referred to as Black), non-Hispanic White (hereafter referred to as White), and other (including American Indian and Alaska Native, multiracial, and other race and ethnicity that were not specified by the patient). Race and ethnicity was included as a variable to learn whether any disparities in outcomes existed among racial and ethnic groups. Because approximately half of the patients in the APCD are missing race and ethnicity data, we deployed the Bayesian Imputed Surname Geocoding algorithm, using the R package wru (R Foundation for Statistical Computing),^13^ to estimate the most likely race and ethnicity category for individuals with missing data (46% total imputed race and ethnicity). Individuals who were estimated to have American Indian and Alaska Native race and ethnicity by the Bayesian Imputed Surname Geocoding algorithm were placed in the other category because the algorithm for American Indian and Alaska Native category was not reliable.^14^

To classify patients as residing in large central metropolitan, large fringe metropolitan, medium metropolitan, small metropolitan, micropolitan, and noncore areas, we linked each patient’s county of residence to the 2013 National Center for Health Statistics urban-rural classification scheme.^15^ These urban-rural classifications were then further collapsed to metropolitan counties, nonmetropolitan counties, or unknown. Insurance type or index year (2015) payer, which patients had for at least 6 months, was identified and categorized as commercial, Medicare Advantage, Medicaid, or dual eligibility (Medicare Advantage and Medicaid). If patients had 2 or more insurance plans in the index year, we assigned to them the plan they had for the longest time. If patients had 2 insurance plans for the same amount of time, we assigned to them a single plan according to the following priority: Medicaid, Medicare Advantage, or commercial.

#### Patient Comorbidities and Pain Diagnoses

We flagged patient comorbidities and pain diagnoses from APCD medical claims in 2013 and 2014. Common chronic and acute pain conditions were flagged using a validated *International Classification of Diseases, Ninth Revision, Clinical Modification* and *International Statistical Classification of Diseases, Tenth Revision, Clinical Modification* crosswalk.^16^ The presence of painful conditions (≥1) was also flagged. We used the R comorbidity package^17^ to identify Elixhauser comorbid conditions^18^ from previously validated definitions.^19^ Comorbidities were classified as 0, 1 to 2, or 3 or more comorbidities. Certain Elixhauser psychiatric comorbidities (alcohol and substance use disorders, depression, and psychosis) were analyzed separately.

#### Prescription Characteristics

We used the US Food and Drug Administration National Drug Code directory to identify opioids and benzodiazepines in the PDMP data set by pharmaceutical class (eTable 2 in the Supplement).^20^ We used an MME conversion file provided by the US Department of Health and Human Services (eFigure in the Supplement).^21^ We excluded opioid prescription fills if they were duplicates (had the same date, drug name, quantity, and days’ supply), had a quantity of less than 4 doses or greater than the 99th percentile, had a number of days’ supply that was less than 0 or greater than 90 days (Figure). All opioid formulations (including patches) were included in the analysis.

Prescription characteristics were categorized into those based on the index opioid prescription and those based on the index period (the first 6 months after opioid initiation, including the month of index prescription). These characteristics were the same for individuals with only 1 prescription given that ongoing use of prescription opioids was not required. Index opioid prescription characteristics included drug name (codeine sulfate, hydrocodone bitartrate, oxycodone hydrochloride, morphine sulfate, tramadol hydrochloride, and other opioids), long- or short-acting release type, and number of days’ supply. Index period characteristics included the number of opioid or benzodiazepine fills, the sum of the opioid dose (quantity multiplied by the strength and the MME factor, which was categorized into <90, 90-149, 150-299, or ≥300 MMEs), 2 flags indicating whether patients received opioids from 3 or more prescribers or pharmacies, and 1 flag for opioid and benzodiazepine overlap in these 6 months.

### Outcomes Measures

An overdose event included both nonfatal and fatal opioid overdoses. Hospital discharge and APCD emergency department claims with diagnoses for opioid poisoning after an index prescription were used to identify first incident nonfatal overdoses (eTable 1 in the Supplement).

Fatal opioid overdoses were identified from the Oregon Vital Records using *International Statistical Classification of Diseases and Related Health Problems, Tenth Revision* underlying cause-of-death codes (X40-X44, X60-X64, X85, and Y10-Y14) with multiple cause-of-death codes (T40.0, T40.1, T40.2, T40.3, T40.4, and T40.6).^22^ We also searched for terms associated with opioid overdose in the literal text fields of the death record, as described previously.^23^

### Statistical Analysis

For descriptive analyses, we generated frequency distributions for patient characteristics (age, sex, race and ethnicity, insurance type [payer in the index year], urbanicity, comorbidities, and pain conditions), index prescription attributes (opioid name, long-acting formulation, and number of days’ supply), and index period variables (number of opioid and benzodiazepine fills, MME sum, opioid and benzodiazepine overlap, and fills from multiple prescribers or pharmacies). We calculated unadjusted overall and characteristic-specific opioid overdose rates per 100 000 person-years.

The hazard ratios (HRs) of patient and prescription-related risk factors of opioid overdose were assessed using a time-dependent Cox proportional hazards regression model. To capture the association of the first 6 months after opioid initiation with risk of overdose, we computed the monthly time-varying prescription variables (MME sum, number of opioid and benzodiazepine fills, opioid and benzodiazepine overlap, and fills from multiple prescribers or pharmacies) cumulatively through the sixth month after which the sixth month’s value was carried over to the rest of the study months. Number of days’ supply and number of opioid prescription fills were not included in the model to avoid an association with the MME variable. We assessed multicollinearity among the risk factors using the variance inflation factor approach and did not find any multicollinearity. For the Cox proportional hazards regression model, the proportional hazards assumption was confirmed by visual assessment of the Schoenfeld residuals plots.

A 2-sided *P* < .05 was considered to be statistically significant. All analyses were conducted with SAS, version 9.3 (SAS Institute Inc), and R, version 1.3 (R Foundation for Statistical Computing). Data analyses were performed from March 1, 2020, to June 15, 2021.

## Results

A total of 236 921 patients began a new opioid episode in 2015. These patients included 133 839 women (56.5%) and 103 082 men (43.5%), 73.6% were White, 41.8% had commercial insurance in 2015, and 72.1% lived in metropolitan counties (Table 1). Nearly a third of the sample had at least 1 pain condition (29.1%), and almost half had 1 or more comorbidities (45.0%). The most prevalent psychiatric condition was depression (12.6%). Patients were followed up for a mean (SD) duration of 33.7 (13.7) months. Of the 236 921 patients, 0.3% experienced opioid overdose.

**Table 1.** Patient Characteristics Associated With Increased Risk of Opioid Overdose Among Opioid-Naive Patients

Characteristic	Total cohort, No. (%)	Opioid overdose, No. (%)	Overdose rate per 100 000 person-years (95% CI)	aHR (95% CI)^a^	*P* value
Overall No.	236 921 (100)	667 (0.3)	120.6 (111.8-130.2)	NA	NA
Age band, y					
18-24	29 006 (12.2)	105 (15.7)	156.4 (129.2-189.3)	1.80 (1.36-2.39)	<.001^b^
25-34	46 122 (19.5)	106 (15.9)	99.8 (82.5-120.7)	0.96 (0.72-1.27)	.77
35-44	39 941 (16.9)	92 (13.8)	96.8 (78.9-118.8)	1 [Reference]	
45-54	39 965 (16.9)	116 (17.4)	119.4 (99.5-143.2)	1.23 (0.93-1.61)	.15
55-64	41 900 (17.7)	104 (15.6)	108.9 (89.9-132.0)	1.24 (0.93-1.66)	.14
65-74	34 458 (14.5)	109 (16.3)	133.1 (110.3-160.6)	1.31 (0.87-1.97)	.20
≥75	5529 (2.3)	35 (5.2)	351.6 (252.4-489.7)	3.22 (1.94-5.36)	<.001^b^
Sex					
Female	133 839 (56.5)	365 (54.7)	114.6 (103.4-126.9)	1 [Reference]	
Male	103 082 (43.5)	302 (45.3)	128.9 (115.2-144.3)	1.29 (1.10-1.51)	.001
Race and ethnicity^c^					
Asian or Pacific Islander	5400 (2.3)	3 (0.4)	22.7 (7.3-70.5)	0.21 (0.07-0.66)	.01
Black	7380 (3.1)	40 (6.0)	231.3 (169.7-315.3)	1.55 (1.12-2.15)	.01
Hispanic	21 696 (9.2)	43 (6.4)	86.9 (64.4-117.2)	0.70 (0.51-0.96)	.03^b^
White	174 335 (73.6)	521 (78.1)	126.6 (116.2-138.0)	1 [Reference]	
Other^d^	3636 (1.5)	16 (2.4)	178.7 (109.5-291.7)	1.03 (0.63-1.71)	.90
Unknown	24 474 (10.3)	44 (6.6)	83.7 (62.3-112.5)	1.18 (0.83-1.67)	.35
Insurance type^e^					
Commercial	98 981 (41.8)	93 (13.9)	40.2 (32.8-49.2)	1 [Reference]	
Dual eligibility	12 738 (5.4)	385 (57.7)	176.3 (159.5-194.8)	4.37 (3.09-6.18)	<.001^b^
Medicaid	93 558 (39.5)	100 (15.0)	134.8 (110.8-164.0)	3.77 (2.97-4.80)	<.001^b^
Medicare Advantage	31 506 (13.3)	89 (13.3)	311.6 (253.1-383.6)	2.18 (1.44-3.31)	<.001^b^
Urban-rural classification					
Metropolitan counties	170 914 (72.1)	57 (8.5)	93.4 (72.0-121.0)	1.51 (1.15-2.00)	.004^b^
Nonmetropolitan counties	26 617 (11.2)	505 (75.7)	125.5 (115.0-136.9)	1 [Reference]	
Unknown	39 390 (16.6)	105 (15.7)	117.6 (97.1-142.4)	1.27 (0.91-1.77)	.17
Pain conditions					
No	168 050 (70.9)	396 (59.4)	102.4 (92.8-113.0)	1 [Reference]	
Yes	68 871 (29.1)	271 (40.6)	163.1 (144.8-183.7)	1.06 (0.89-1.25)	.52
Psychiatric conditions					
Alcohol use disorder					
No	231 431 (97.7)	537 (80.5)	117.1 (107.6-127.5)	1 [Reference]	
Yes	5490 (2.3)	41 (6.1)	314.5 (231.6-427.1)	1.01 (0.72-1.41)	.97
Depression					
No	207 179 (87.5)	404 (60.6)	101.5 (92.0-111.8)	1 [Reference]	
Yes	29 742 (12.6)	174 (26.1)	237.4 (204.6-275.4)	1.26 (1.03-1.55)	.03^b^
Psychosis					
No	233 241 (98.5)	537 (80.5)	116.1 (106.6-126.3)	1 [Reference]	
Yes	3680 (1.6)	41 (6.1)	466.4 (343.4-633.4)	1.18 (0.84-1.67)	.34
Substance use disorder					
No	230 472 (97.3)	477 (71.5)	104.7 (95.8-114.6)	1 [Reference]	
Yes	6449 (2.7)	101 (15.1)	626.4 (515.4-761.3)	2.74 (2.15-3.50)	<.001^b^
No. of comorbidities					
0	130 093 (54.9)	218 (32.7)	73.2 (64.1-83.6)	1 [Reference]	
1-2	84 018 (35.5)	280 (42.0)	138.2 (122.9-155.4)	1.32 (1.08-1.62)	.01^b^
≥3	22 810 (9.6)	169 (25.3)	321.9 (276.8-374.3)	1.90 (1.42-2.53)	<.001^b^

Abbreviations: aHR, adjusted hazard ratio; NA, not applicable.

^a^ Adjusted for index prescription drug, long-acting formulation, number of benzodiazepine prescriptions, opioid and benzodiazepine overlap, sum of morphine milligram equivalents, and prescription from 3 or more prescribers or pharmacies.

^b^ Statistically significant at *P* < .05.

^c^ Race and ethnicity data were obtained from the Oregon All Payer All Claims Data enrollment files. Missing data for approximately half of the cohort were imputed using Bayesian Imputed Surname Geocoding algorithm.

^d^ Other race and ethnicity included American Indian and Alaska Native, multiracial, and other categories that were not specified by the patient.

^e^ Index year (2015) payer.

### Characteristics of the Index Prescription and Index Period

Most patients received hydrocodone (59.2%) as their initial opioid prescription, followed by oxycodone (26.1%). Other less common initiation opioids included tramadol (7.8%) and codeine (4.9%); few were long-acting opioids (0.3%). Most prescriptions had 7 days’ supply or less (Table 2).

**Table 2.** Prescription Characteristics Associated With Increased Risk of Opioid Overdose Among Opioid-Naive Patients

Characteristic	Total cohort, No. (%)	Overdose, No. (%)	Overdose rate per 100 000 person-years (95% CI)	aHR (95% CI)^a^	*P* value
**Index prescription**					
Opioid name					
Codeine sulfate	11 536 (4.9)	21 (3.1)	76.0 (49.5-116.5)	1 [Reference]	
Hydrocodone bitartrate	140 362 (59.2)	336 (50.4)	101.8 (91.5-113.3)	1.31 (0.84-2.04)	.23
Morphine sulfate	2283 (1.0)	5 (0.7)	246.8 (102.7-592.9)	0.90 (0.33-2.48)	.84
Other opioids	2560 (1.1)	23 (3.4)	403.9 (268.4-607.8)	1.68 (1.07-2.66)	.03^b^
Oxycodone hydrochloride	61 721 (26.1)	210 (31.5)	145.1 (126.7-166.1)	1.70 (1.04-2.77)	.04^b^
Tramadol hydrochloride	18 459 (7.8)	72 (10.8)	167.9 (133.2-211.5)	2.80 (1.34-5.84)	.01^b^
Long-acting formulation					
No	236 136 (99.7)	655 (98.2)	118.8 (110.1-128.3)	1 [Reference]	
Yes	785 (0.3)	12 (1.8)	734.8 (417.3-1294.0)	1.24 (0.54-2.82)	.61
No. of days’ supply, d^c^					
≤7	190 900 (80.6)	516 (77.4)	108.9 (99.9-118.7)	NA	
>7	34 439 (14.5)	151 (22.6)	191.3 (163.1-224.4)	NA	
**Index period**					
No. of opioid fills^c^					
1-2	193 961 (81.9)	434 (65.1)	95.6 (87.0-105.0)	NA	
3-5	32 191 (13.6)	138 (20.7)	184.5 (156.1-218.0)	NA	
≥6	10 769 (4.6)	95 (14.2)	395.3 (323.3-483.4)	NA	
MME sum					
<90	23 423 (9.9)	31 (4.6)	59.1 (41.6-84.1)	1 [Reference]	
90-149	26 774 (11.3)	32 (4.8)	52.5 (37.1-74.2)	0.98 (0.74-1.30)	.90
150-299	54 776 (23.1)	88 (13.2)	68.7 (55.8-84.7)	0.93 (0.72-1.20)	.57
300+	131 948 (55.7)	516 (77.4)	165.7 (152.0-180.6)	1.24 (0.96-1.60)	.10
No. of benzodiazepine fills					
0	206 217 (87.0)	500 (75.0)	103.1 (94.5-112.6)	1 [Reference]	
≥1	30 704 (13.0)	167 (25.0)	245.7 (211.1-285.9)	1.06 (1.01-1.11)	.01^b^
Opioid and benzodiazepine overlap					
No	219 349 (92.6)	566 (84.9)	109.6 (101.0-119.0)	1 [Reference]	
Yes	17 572 (7.4)	101 (15.1)	276.1 (227.2-335.5)	2.11 (1.70-2.62)	<.001^b^
Fills from ≥3 pharmacies					
No	230 834 (97.4)	614 (92.1)	113.9 (105.2-123.2)	1 [Reference]	
Yes	6087 (2.6)	53 (7.9)	387.5 (296.1-507.2)	1.38 (1.09-1.75)	.01^b^
Fills from ≥3 prescribers					
No	213 886 (90.3)	514 (77.1)	102.9 (94.3-112.1)	1 [Reference]	
Yes	23 035 (9.7)	153 (22.9)	287.9 (245.7-337.4)	1.37 (0.97-1.94)	.08

Abbreviations: aHR, adjusted hazard ratio; MME, morphine milligram equivalent; NA, not applicable.

^a^ Adjusted for age, sex, race and ethnicity, urban-rural classification, insurance type or payer of index year, pain conditions, mental health conditions (substance use disorder, alcohol use disorder, psychosis, and depression), and number of comorbidities.

^b^ Statistically significant at *P* < .05.

^c^ Number of days’ supply and number of opioid prescriptions not included in the Cox proportional hazards regression model.

During the 6-month index period, most patients (81.9%) had 1 or 2 opioid prescriptions. More than half of the patients in the cohort (55.7%) had cumulative doses of 300 MME or more, 2.6% of patients received prescriptions from 3 or more pharmacies, and 9.7% received opioid prescriptions from 3 or more prescribers. In addition, 13.0% of patients had at least 1 benzodiazepine prescription, and 7.4% received overlapping opioid and benzodiazepine prescriptions in the index period (Table 2).

### Opioid Overdose Rates and Risk Factors

The overall unadjusted rate of fatal or nonfatal opioid overdose was 120.6 (95% CI, 111.8-130.2) per 100 000 person-years (Table 1); however, some patients had much higher opioid overdose rates. Higher rates were seen in patients with a history of comorbid substance use disorder (626.4 [95% CI, 515.4-761.3] per 100 000 person-years) with comorbid psychosis (466.4 [95% CI, 343.4-633.4] per 100 000 person-years) whose index prescription was long-acting (734.8 [95% CI, 417.3-1294.0] per 100 000 person-years) and who, in the 6-month index period, filled 6 or more opioid prescriptions (395.3 [95% CI, 323.3-483.4] per 100 000 person-years) or filled opioid prescriptions from 3 or more pharmacies (387.5 [95% CI, 296.1-507.2] per 100 000 person-years).

Factors associated with opioid overdose risk are shown in Tables 1 and 2. Significant findings included that risk was higher among individuals 75 years or older (adjusted HR [aHR], 3.22; 95% CI, 1.94-5.36) and aged 18 to 24 years (aHR, 1.80; 95% CI, 1.36-2.39) compared with those aged 35 to 44 years, men (aHR, 1.29; 95% CI, 1.10-1.51) compared with women; Black patients (aHR, 1.55; 95% CI, 1.12-2.15) compared with White patients; those with dual eligibility for Medicaid and Medicare Advantage (aHR, 4.37; 95% CI, 3.09-6.18), with Medicaid (aHR, 3.77; 95% CI, 2.97-4.80), or with Medicare Advantage (aHR, 2.18; 95% CI, 1.44-3.31) compared with commercial insurance; and those with comorbid substance use disorder (aHR, 2.74; 95% CI, 2.15-3.50), those with depression (aHR, 1.26; 95% CI, 1.03-1.55), and those with 1 or 2 comorbidities (aHR, 1.32; 95% CI, 1.08-1.62) or 3 or more comorbidities (aHR, 1.90; 95% CI, 1.42-2.53). Opioid-naive patients were also at an increased overdose risk if they filled oxycodone (aHR, 1.70; 95% CI, 1.04-2.77) or tramadol (aHR, 2.80; 95% CI, 1.34-5.84) as an index prescription compared with codeine, and if in the index period they filled benzodiazepines (aHR, 1.06; 95% CI, 1.01-1.11), concurrent opioids and benzodiazepines (aHR, 2.11; 95% CI, 1.70-2.62), or opioids from 3 or more pharmacies (aHR, 1.38; 95% CI, 1.09-1.75).

## Discussion

This study found several patient and prescription-related risk factors that can provide guidance to clinicians, when either writing the initial opioid prescription or prescribing over the next several months, in the early period of a patient’s opioid prescription experience. These risk factors may be considered by prescribers to identify opioid-naive patients who may be most at risk for harm; to inform patient counseling and monitoring if opioids are prescribed; and to provide additional information on safe use, storage, and disposal of opioids and on signs of overdose.

The first consideration should be patient-level factors. In the present cohort, those who were at the highest adjusted risk for subsequent overdose were older (≥75 years) and younger (18-24 years) individuals. The fact that older patients had an HR of 3.22 compared with the reference group (aged 35-44 years) is concerning because opioid prescribing to older patients occurs frequently, and recent data have shown an increase of opioid-related hospitalizations among older patients.^24^ This finding differs from national data showing that, in 2019, individuals 65 years or older had the lowest rates of drug overdose deaths compared with other age groups^25^ and may reflect the inclusion criteria for the cohort, which required an incident opioid prescription. For younger patients, the morbidity and mortality that were associated with heroin and fentanyl use were substantially higher than in the older age groups,^26^ and the initial opioid prescription may be an individual’s first experience with opioids, increasing the risk of using illicit opioids.

The second consideration in prescribing opioids should be the race and ethnicity of the patient.^27^ In this study, we found a significantly lower risk of opioid overdose among Asian or Pacific Islander individuals compared with White patients. Hispanic patients were also less likely to be at risk for overdose. Previous studies have found that opioid prescriptions are less likely to be given to Asian or Pacific Islander and Hispanic groups in the first place.^28,29^ Conversely, Black individuals had a significantly higher risk of overdose, which is particularly alarming given the disparities seen in overdose deaths during the COVID-19 pandemic.^30^ Nevertheless, given that the state of Oregon has a predominantly White population and that we imputed some race and ethnicity data, further study is needed that would allow definitive conclusions to be drawn.

The third consideration should be the patient insurance type, which was also associated with overdose risk. Patients with Medicaid had an almost 4-fold increased risk of overdose compared with the commercially insured population, whereas those who were dually eligible for Medicaid and Medicare Advantage had an even higher risk. A study that evaluated patients with opioid use disorder who had Medicaid coverage found that those with a co-occurring substance use disorder were even more at risk for opioid-related adverse events.^31^ Another study in Maryland found that Medicaid and Medicare insurance was a strong risk factor for opioid overdose.^32^ A study in Washington state reported that the risk for prescription opioid overdose death was about 6 times higher in Medicaid enrollees than in the non-Medicaid population.^33,34^ Given that access to opioid agonist treatment remains low,^35^ the findings from this study support the need for more addiction treatment resources for publicly insured patients.

The fourth consideration should be medical and psychiatric comorbidities when prescribing opioids to a previously opioid-naive patient. Individuals with 3 or more comorbidities had an HR of 1.90, and previous research conducted in a large health system reported that cardiovascular disease, diabetes, and cancer were associated with overdose.^36^ The psychiatric diagnoses of depression and previous substance use disorder were also associated with increased risk of overdose. We believe that the present study further supports the association between psychiatric risk factors and opioid overdose.^37,38^ The findings of patient-level characteristics are consistent with the risk factors that are present in commonly used screening tools, such as the Opioid Risk Tool.^39^ Despite developing in a much smaller cohort of patients and with a different end point (opioid-related aberrant behaviors), the high-risk features used in the Opioid Risk Tool, such as male sex, history of substance use disorder, and other concurrent psychiatric conditions, were also found in this study, suggesting that the results of this study could be converted into a more comprehensive risk assessment scoring tool that incorporates other social determinants of health such as urbanicity of residence and insurance type.^40^

The fifth consideration should be prescription-level characteristics. Compared with codeine, oxycodone and tramadol that were used as the initial opioid prescriptions were associated with an increased risk, whereas hydrocodone and morphine were not statistically different. These findings have important implications for prescribers because risk is not only isolated to the decision to prescribe an opioid but also is dependent on which opioid is chosen as the initial prescription. Although the incidence of overdose was high among individuals who were prescribed long-acting formulations, after analyses were controlled and adjusted for MMEs, the aHR of overdose risk was not higher. In addition, incidence of overdose was not associated with varying levels of MME that were received in the first 6 months, which may indicate that patient factors may be more important than the strength of the opioids prescribed. These are both novel findings. The risk of concurrent prescription of opioids and benzodiazepines cannot be understated. The aHR for patients who received both classes of medications in the index period was 2.11, which corroborates the findings from other research.^41^

The findings from this study may be used by researchers to develop clinical decision-making tools as well as by policymakers and insurers to provide additional prevention and addiction treatment resources to individuals who are most vulnerable to opioid overdose.

### Limitations

This study has some limitations. First, it is a retrospective analysis of administrative data, and not a prospective data collection. Missing data on race and ethnicity, for example, were not missing at random and had to be imputed, and thus may be imprecise. Additional patient-level factors that were not present in the data, such as educational level, income, and other social determinants of health, could not be accounted for and may be reflected by surrogate findings, such as the differences between publicly vs privately insured individuals. Second, we included patients with Medicare Advantage, but we did not include patients with standard Medicare fee-for-service coverage or patients without insurance. However, we did not exclude certain diagnoses, such as terminal cancer. Third, because prescribing practices and the illicit opioid supply have changed dramatically since the study was conducted, the clinical applicability of these findings may be different today. We were unable to use methadone or naltrexone as a marker of opioid use disorder because they do not appear in the PDMP data for this indication. Oregon is a unique state, and the results in this setting may not apply to other settings.

## Conclusions

In this cohort study, using a comprehensive database and time-varying analysis model, we identified patient factors (aged ≥75 years, male sex, Medicaid or Medicare Advantage insurance, comorbid substance use disorder or depression, and medical comorbidities) and prescription-related factors (oxycodone or tramadol as the initial prescription, concurrent use of benzodiazepines, and filling opioid prescriptions from ≥3 pharmacies) that were associated with opioid overdose. These risk factors may be used by prescribers to identify opioid-naive patients; to inform opioid counseling and monitoring; and to educate about safe opioid use, storage, and disposal and signs of overdose. These findings may also be used by researchers to develop clinical decision-making tools, and policymakers and insurers may use the data to provide opioid prevention and treatment resources to individuals who are at greatest risk for opioid overdose.

## Notes

Supplement. **eTable 1.** *International Classification of Diseases (ICD)* Version 9 or 10 Diagnosis Codes Used to Ascertain Emergency Department or Inpatient Admission for Opioid-Related Overdose/Poisoning or Presence of Opioid Use Disorder **eTable 2.** National Drug Codes for Benzodiazepines and Opioids Used for the Study, Including Morphine Milligram Conversion Factors, Where Applicable **eFigure.** US Department of Health and Human Services Opioid Oral Morphine Milligram Equivalent (MME) Conversion Factors Table for Prescription Drug Coverage
