# Supplementary material for: Factors Associated With Opioid Overdose After an Initial Opioid Prescription
Source: JAMA Netw Open. 2022 Jan 28;5(1):e2145691. doi: 10.1001/jamanetworkopen.2021.45691 (PMC8800077; doi:10.1001/jamanetworkopen.2021.45691)
Supplement: Supplement. — eTable 1. International Classification of Diseases (ICD) Version 9 or 10 Diagnosis Codes Used to Ascertain Emergency Department or Inpatient Admission for Opioid-Related Overdose/Poisoning or Presence of Opioid Use Disorder eTable 2. National Drug Codes for Benzodiazepines and Opioids Used for the Study, Including Morphine Milligram Conversion Factors, Where Applicable eFigure. US Department of Health and Human Services Opioid Oral Morphine Milligram Equivalent (MME) Conversion Factors Table for Prescription Drug Coverage [file jamanetwopen-e2145691-s001.pdf]

Supplementary Online Content

Weiner SG, El Ibrahimi S, Hendricks MA, et al. Factors associated with opioid overdose after an initial opioid prescription. *JAMA Netw Open*. 2022;5(1):e2145691. doi:10.1001/jamanetworkopen.2021.45691

eTable 1. *International Classification of Diseases (ICD)* Version 9 or 10 Diagnosis Codes Used to Ascertain Emergency Department or Inpatient Admission for Opioid-Related Overdose/Poisoning or Presence of Opioid Use Disorder

eTable 2. National Drug Codes for Benzodiazepines and Opioids Used for the Study, Including Morphine Milligram Conversion Factors, Where Applicable

eFigure. US Department of Health and Human Services Opioid Oral Morphine Milligram Equivalent (MME) Conversion Factors Table for Prescription Drug Coverage

This supplementary material has been provided by the authors to give readers additional information about their work.

eTable1. *International Classification of Diseases (ICD)* Version 9 or 10 Diagnosis Codes Used to Ascertain Emergency Department or Inpatient Admission for Opioid-Related Overdose/Poisoning or Presence of Opioid Use Disorder

**Overdose/Poisoning Codes**		
ICD-9	*Poisoning by:*	
	opium	96500
	heroin	96501
	methadone	96502
	other opiates and related narcotics	96509
	Accidental poisoning by:	
	heroin	E8500
	methadone	E8501
	other opiates and related narcotics	E8502
ICD-10	*Poisoning by:*	
	opium	T40.0
	heroin	T40.1
	natural or semi-synthetic opioids	T40.2
	methadone	T40.3
	synthetic opioids, other than methadone	T40.4
	other and unspecified narcotics	T40.6

**Opioid Use Disorder Codes**		
ICD-9	Opioid dependence	304.00–304.03
	Opioid or other drug dependence	304.70–304.73
	Nondependent drug abuse	305.50–305.53
ICD-10	Opioid abuse, dependence	F11.1x – F11.2x
	Opioid use	F11.9x

© 2022 Weiner SG et al. *JAMA Network Open.*

eTable 2. National Drug Codes for Benzodiazepines and Opioids Used for the Study, Including Morphine Milligram Conversion Factors, Where Applicable

NDC	DC_Numer	PRODNME	GENNME	laster_Forı	Class	Drug	ıgShortAct	EAClassCo	ngth_Per_
005401761	54017613	HINE HYDI	ɔhine Hydr	Tablet	Opioid	prenorphi	LA	3	2
005401771	54017713	HINE HYDI	ɔhine Hydr	Tablet	Opioid	prenorphi	LA	3	8
005401881	54018813	RPHINE-N.	ɔrphine/na	Tablet	Opioid	prenorphi	LA	3	2
005401891	54018913	RPHINE-N.	ɔrphine/na	Tablet	Opioid	prenorphi	LA	3	8
009336002	93360021	›RENORPH	prenorphi	ˑxtended F	Opioid	prenorphi	LA	3	5
009336004	93360040	›RENORPH	prenorphi	ˑxtended F	Opioid	prenorphi	LA	3	5
009336012	93360121	›RENORPH	prenorphi	ˑxtended F	Opioid	prenorphi	LA	3	10
009336014	93360140	›RENORPH	prenorphi	ˑxtended F	Opioid	prenorphi	LA	3	10
009336022	93360221	›RENORPH	prenorphi	ˑxtended F	Opioid	prenorphi	LA	3	15
009336024	93360240	›RENORPH	prenorphi	ˑxtended F	Opioid	prenorphi	LA	3	15
009336032	93360321	›RENORPH	prenorphi	ˑxtended F	Opioid	prenorphi	LA	3	20
009336034	93360340	›RENORPH	prenorphi	ˑxtended F	Opioid	prenorphi	LA	3	20
009353785	93537856	HINE HYDI	ɔhine Hydr	Tablet	Opioid	prenorphi	LA	3	2
009353795	93537956	HINE HYDI	ɔhine Hydr	Tablet	Opioid	prenorphi	LA	3	8
009357205	93572056	RPHINE-N.	ɔrphine/na	Tablet	Opioid	prenorphi	LA	3	2
009357215	93572156	RPHINE-N.	ɔrphine/na	Tablet	Opioid	prenorphi	LA	3	8
022831530	2.28E+08	HINE HYDI	ɔhine Hydr	Tablet	Opioid	prenorphi	LA	3	8
022831540	2.28E+08	RPHINE-N.	ɔrphine/na	Tablet	Opioid	prenorphi	LA	3	2
022831547	2.28E+08	RPHINE-N.	ɔrphine/na	Tablet	Opioid	prenorphi	LA	3	2
022831550	2.28E+08	RPHINE-N.	ɔrphine/na	Tablet	Opioid	prenorphi	LA	3	8
022831557	2.28E+08	RPHINE-N.	ɔrphine/na	Tablet	Opioid	prenorphi	LA	3	8
022831560	2.28E+08	HINE HYDI	ɔhine Hydr	Tablet	Opioid	prenorphi	LA	3	2
037809239	3.78E+08	HINE HYDI	ɔhine Hydr	Tablet	Opioid	prenorphi	LA	3	2
037809249	3.78E+08	HINE HYDI	ɔhine Hydr	Tablet	Opioid	prenorphi	LA	3	8
040619230	4.06E+08	RPHINE-N.	ɔrphine/na	Tablet	Opioid	prenorphi	LA	3	2
040619240	4.06E+08	RPHINE-N.	ɔrphine/na	Tablet	Opioid	prenorphi	LA	3	8

© 2022 Weiner SG et al. *JAMA Network Open.*

eFigure. US Department of Health and Human Services Opioid Oral Morphine Milligram Equivalent (MME) Conversion Factors Table for Prescription Drug Coverage

Opioid Oral Morphine Milligram Equivalent (MME) Conversion Factors[i,ii]

Type of Opioid (strength units)	MME Conversion Factor
Buprenorphine film/tablet[iii] (mg)	
Buprenorphine patch[iii] (mcg/hr)	
Buprenorphine film [iii] (mcg)	
Butorphanol (mg)	7
Codeine (mg)	0.15
Dihydrocodeine (mg)	0.25
Fentanyl buccal or SL tablets, or lozenge/troche[iv] (mcg)	0.13
Fentanyl film or oral spray[v] (mcg)	0.18
Fentanyl nasal spray[vi] (mcg)	0.16
Fentanyl patch[vii] (mcg)	7.2
Hydrocodone (mg)	1
Hydromorphone (mg)	4
Levorphanol tartrate (mg)	11
Meperidine hydrochloride (mg)	0.1
Methadone[viii] (mg)	3
>0, <= 20	4
>20, <=40	8
>40, <=60	10
>60	12
Morphine (mg)	1
Opium (mg)	1
Oxycodone (mg)	1.5
Oxymorphone (mg)	3
Pentazocine (mg)	0.37
Tapentadol[ix] (mg)	0.4
Tramadol (mg)	0.1

[i] The MME conversion factor is intended only for analytic purposes where prescription data are used to calculate daily MME. Use the formula: Strength per Unit X (Number of Units/ Days Supply) X MME conversion factor = MME/Day. This value does not constitute clinical guidance or recommendations for converting patients from one form of opioid analgesic to another. Please consult the manufacturer's full prescribing information for such guidance. Use of this file for the purposes of any clinical decision-making warrants caution. This is particularly true with regard to methadone (see viii below).

[ii] National Center for Injury Prevention and Control. CDC compilation of benzodiazepines, muscle relaxants, stimulants, zolpidem, and opioid analgesics with oral morphine milligram equivalent conversion factors, 2017 version. Atlanta, GA: Centers for Disease Control and Prevention; Available at https://www.cdc.gov/drugoverdose/resources/data.html . For more information, send an email to Mbohm@cdc.gov.

[iii] Buprenorphine products are listed but do not have an associated MME conversion factor. These buprenorphine products, as partial opioid agonists, are not expected to be associated with overdose risk in the same dose-dependent manner as doses for

© 2022 Weiner SG et al. *JAMA Network Open.*

full agonist opioids. The conversion factors for drugs prescribed or provided as part of medication-assisted treatment for opioid use disorder should not be used to benchmark against dosage thresholds meant for opioids prescribed for pain.

[iv] The MME conversion factor for fentanyl buccal tablets, sublingual tablets, and lozenges/troche is 0.13. This conversion factor should be multiplied by the number of micrograms in a given tablet or lozenge/troche.

[v] The MME conversion factor for fentanyl film and oral spray is 0.18. This reflects a 40% greater bioavailability for films compared to lozenges/tablets and 38% greater bioavailability for oral sprays compared to lozenges/tablets.

[vi] The MME conversion factor for fentanyl nasal spray is 0.16, which reflects a 20% greater bioavailability for sprays compared to lozenges/tablets.

[vii] The MME conversion factor for fentanyl patches is based on the assumption that one milligram of parenteral fentanyl is equivalent to 100 milligrams of oral morphine and that one patch delivers the dispensed micrograms per hour over a 24 hour day.
Example: 25 ug/hr fentanyl patch X 24 hrs = 600 ug/day fentanyl = 60 mg/day oral morphine milligram equivalent.
In other words, the conversion factor not accounting for days of use would be 60/25 or 2.4.
However, since the fentanyl patch remains in place for 3 days, we have multiplied the conversion factor by 3 (2.4 X 3 = 7.2). In this example, MME/day for ten 25 µg/hr fentanyl patches dispensed for use over 30 days would work out as follows:
Example: 25 ug/hr fentanyl patch X (10 patches/30 days) X 7.2 = 60 MME/day. Please note that because this allowance has been made based on the typical dosage of one fentanyl patch per 3 days, you should first change all Days Supply in your prescription data to follow this standard, i.e., Days Supply for fentanyl patches= # of patches X 3.

[viii] The CDC MME conversion factor to calculate morphine milligram equivalents of methadone is 3. Calculating MME for methadone in clinical practice often involves a sliding-scale approach whereby the conversion factor increases with increasing dose since the conversion factor of 3 for methadone could underestimate MME for a given patient. CMS uses this conversion factor when analyzing Medicare population opioid use. CMS uses the graduated methadone MME conversion factors to calculate MME within the Overutilization Monitoring System (OMS) for identifying and reporting potential opioid overutilizers. https://www.cdc.gov/drugoverdose/pdf/calculating_total_daily_dose-a.pdf.

[ix] Tapentadol is a mu receptor agonist and norepinephrine reuptake inhibitor. Oral MMEs are based on degree of mu receptor agonist activity, but it is unknown if this drug is associated with overdose in the same dose-dependent manner as observed with medications that are solely mu receptor agonists.

© 2022 Weiner SG et al. *JAMA Network Open.*
